# Ligation Strategies for Targeting Liposomal Nanocarriers

**DOI:** 10.1155/2014/129458

**Published:** 2014-07-14

**Authors:** Patricia Marqués-Gallego, Anton I. P. M. de Kroon

**Affiliations:** Membrane Biochemistry & Biophysics, Bijvoet Center for Biomolecular Research and Institute of Biomembranes, Utrecht University, Padualaan 8, H.R. Kruyt Building, 3584 CH Utrecht, The Netherlands

## Abstract

Liposomes have been exploited for pharmaceutical purposes, including diagnostic imaging and drug and gene delivery. The versatility of liposomes as drug carriers has been demonstrated by a variety of clinically approved formulations. Since liposomes were first reported, research of liposomal formulations has progressed to produce improved delivery systems. One example of this progress is stealth liposomes, so called because they are equipped with a PEGylated coating of the liposome bilayer, leading to prolonged blood circulation and improved biodistribution of the liposomal carrier. A growing research area focuses on the preparation of liposomes with the ability of targeting specific tissues. Several strategies to prepare liposomes with active targeting ligands have been developed over the last decades. Herein, several strategies for the functionalization of liposomes are concisely summarized, with emphasis on recently developed technologies for the covalent conjugation of targeting ligands to liposomes.

## 1. Introduction

Their biocompatibility, biodegradability, low toxicity, and capacity to encapsulate a vast variety of drugs make liposomes highly attractive as therapeutic drug carriers. Since phospholipid-based liposomes were first described [1], the targeting and delivery of therapeutic drugs and imaging agents using liposome nanocarriers have made significant advances [2–10]. Progress in liposomal design is leading to improved systems for therapeutic as well as diagnostic applications [5, 11, 12]. Liposomes are increasingly being developed towards contrast-enhanced, cellular, and molecular MRI diagnostic agents [13]. More importantly, clinical studies have confirmed the therapeutic properties of liposomes with the introduction of liposomal drug formulations for the treatment of several diseases [9, 14, 15].

The integrity and stability of liposomes are highly dependent on their chemical composition [16, 17]. The size and number of bilayers of liposomes are controlled by the method of preparation giving rise to multilamellar or unilamellar vesicles of a defined diameter [6]. The liposome size is a highly relevant parameter, influencing its circulation in the body [18, 19]. After intravenous administration, liposomes are often rapidly cleared from the blood [20], and a large amount of liposomes ends up in the liver and spleen [21], which is a drawback of their therapeutic use. Therefore, many studies have been conducted to optimize the therapeutic profile of liposomes, aiming at the improvement of their stability* in vivo* [8, 17]. This led to the introduction of liposomal systems with polyethylene glycol (PEG) incorporated in the outer leaflet of the liposome. PEG groups help to avoid the reticuloendothelial system (RES), resulting in a longer half-life of the nanocarrier in circulation [22].

Doxorubicin entrapped in PEG liposomes (Doxil) was approved by the FDA in the early 90s and presents the first example of an FDA approved nanodrug [23]. The Doxil formulation presents three main features; first, it displays a prolonged drug circulation time due to the use of polyethylene glycol on the surface of the liposomes [21, 24]. Second, it shows good stability of the liposomes for remote loading of doxorubicin by a transmembrane ammonium sulfate gradient, achieving the high drug loading required for its therapeutic effect (routine i.v. doxorubicin is 10–50 mg/m^2^) [25]. Third, the hydrogenated soy bean phosphatidylcholine (HSPC) and cholesterol composition of the lipid bilayer in Doxil formulation render a “liquid ordered” phase with a high transition temperature (*T*_*m*_), stabilizing the liposomes and avoiding undesired release of the drug [26]. All these parameters were optimized over the course of two decades of research of the Doxil formulation, reviewed recently by one of its creators [23].

Today the commercial availability of a vast variety of head group-modified lipids has encouraged researchers to develop more sophisticated liposomal nanocarriers with the ultimate goal of improving clinical results. For instance, the so-called immunoliposomes aim to increase the accumulation of the therapeutic agent at the diseased tissue using targeting moieties attached to the outer leaflet of the liposomal membrane [27]. A variety of strategies to modify the liposome surface have been reported over the last decades [7]. Generally, two main approaches have been chosen to functionalize the liposome surface. One approach is to first attach the targeting ligand of interest to a lipid and then mix the functionalized lipid with other lipid components to prepare liposomes [28, 29]. This method, however, is not convenient for attaching ligands of a large size that complicates the synthesis of the functionalized lipid and its characterization and for ligands that lose their active conformation in organic solvents. The synthesis of modified lipids is more convenient for small ligands that are easy to manipulate and characterize. Alternatively, functionalization of preformed liposomes with the desired targeting ligand is performed on the surface of the liposome [30–37]. For this approach, head group-modified lipids with a polyethylene glycol spacer functionalized at the end with amine, carboxylic acid, thiol, or maleimide groups offer excellent opportunities. These PEGylated lipids are generally incorporated at a low percentage (5–10%) of total lipids in the liposomes. Part of the functional groups is exposed on the outside surface of the liposomes. Modification of the liposomal surface is achieved by performing the chemistry (amide conjugation, hydrazone bond, thioester, or disulfide bridge formation) on the preformed liposomes. The advantage of this approach is that only small amounts of targeting ligand are required, which is particularly useful for the attachment of large macromolecules such as proteins [38]. Finally, a variation of the two main approaches, that is, postinsertion of the functionalized lipid in preformed liposomes, was investigated in the early 90s [39–41]. This approach is however challenging, as the optimal conditions to afford the final insertion product may vary depending on the formulation of the liposome bilayer. The postinsertion of a new lipid is expected to alter the membrane of the liposomes, which may cause leakage of the entrapped agent [40].

This review will describe recent approaches for the development of targeted liposomes. The choice of the strategy to covalently attach the desired targeting moiety to the liposome is mostly determined by the functional groups available on the targeting molecule and the liposome. The conjugation between the liposome and the targeting molecule may affect liposome stability. Liposome bilayer composition, size, and curvature are important parameters to consider when designing the liposomal system and its method of preparation [17, 42]. Even though the stability of liposomes in biological systems is a crucial parameter for serving as efficient therapeutic or imaging agents, many studies do not report on it.

Well-established chemical reactions have been applied to attach different moieties to the lipid or to the surface of preformed liposomes, including amine-carboxylic acid conjugation [30], disulfide bridge formation [43], hydrazone bond formation [44], and the thiol-maleimide addition reaction yielding a thioester bond [45, 46]. More innovative strategies to modify liposomes based on chemical reactions developed over the last decade, such as bioorthogonal chemistry, have been reported more recently [47–52]. The aim of this review is to summarize and give an update on the strategies to functionalize the surface of liposomes for improved targeting of drug-carrier nanosystems.

## 2. Overview of Strategies for Ligand Ligation

### 2.1. Preparation of the Targeted Lipid prior to Incorporation in Liposomes

The synthesis of the targeted lipid prior to the preparation of the liposome has several advantages such as full characterization of the targeted lipid. For instance, doxorubicin-loaded liposomes containing the anisamide ligand on the surface for targeting sigma receptors were successfully made using this approach [29]. Sigma receptors are membrane-bound proteins, which are overexpressed in several types of cancer, and for which anisamide displays good affinity [53].

For targeting doxorubicin-loaded liposomes to the sigma receptors, Banerjee et al. [29] prepared and fully characterized an anisamide-modified lipid. Two different approaches with two different anisamide derivatives were described for conjugating the targeting ligand to the DSPE-PEG-amine phospholipid. In the first approach, 7-[2-(4-methoxybenzylamino)-ethylamino]heptanoic acid was conjugated to the DSPE-PEG-amine lipid using standard DCC/DMAP chemistry for amine-carboxyl conjugation. The second approach yielded the N-alkylated lipid using an N-(2-bromoethyl)-4-methoxybenzamide derivative and DSPE-PEG-amine. The authors showed that the synthesis of the anisamide-lipid by the second approach has 10-fold greater yield than the standard amine-carboxyl coupling. The anisamide-lipid was mixed with the other lipids (POPC and cholesterol) at the desired concentration to form liposomes in citrate buffer (pH 4.0). Controlling the amount of targeting ligand included in the liposomes is an additional advantage of this approach. Doxorubicin was then loaded into the liposomes using the transmembrane pH gradient (acidic inside) according to the remote loading technique developed by Mayer et al. [54]. The formulation showed promising results in the* in vivo* treatment of DU-145 tumors in nude mice. The partitioning of the ligated lipid between the exterior and the interior of the liposome upon liposome formation must be considered, with an estimated 50% of the targeting ligand entrapped in the inner side of the bilayer. Positive results obtained in* in vitro* studies targeting sigma receptors suggested sufficient targeting ligand on the outer side of the liposome bilayer. Possible leakage of doxorubicin from the liposomes induced by the presence of the ligand was not examined.

Recently, a high-affinity peptide ATP_EDB_-conjugated liposome was reported for targeting the extra-domain B (EDB) in glioma therapy [55]. The peptide-lipid APT_EDB_-PEG_2000_-DSPE was prepared and characterized prior to preparing the liposomes [46]. The ATP_EDB_ peptide containing a reactive cysteine at the N-terminus was conjugated to DSPE-PEG_2000_-maleimide phospholipid as confirmed by MALDI-TOF. APT_EDB_-liposomes loaded with doxorubicin were subsequently investigated. Doxorubicin was encapsulated by the addition of the drug during the hydration step.* In vitro* studies of the resulting doxorubicin-loaded liposomes targeting the EDB domain were performed by fluorescence microscopy, monitoring doxorubicin red fluorescence. The targeting ability of this liposomes was confirmed after incubation with GL26 cells (EDB positive) and PC3 cells (EDB negative), with an increase of red fluorescence intensity in GL26 cells, whereas no increase was observed in PC3 cells. Antitumor efficacy of the APT_EDB_-liposomes loaded with doxorubicin was investigated using a GL26 tumor allograft animal model. Tumor size was reduced by 55% as compared to nontreated animals, whereas only 20% size reduction was observed in the animals treated with free doxorubicin [46].

### 2.2. Preformed Liposomes Functionalization

Postfunctionalization of liposomes with small molecules [29], imaging agents [12], peptides, large proteins, or antibodies via direct conjugation to the surface of preformed liposomes has been extensively explored since the first description of liposomes [8, 38]. The main disadvantage of this approach is that the reaction conditions for attaching the targeting ligand may cause destabilization of the liposomes. Modification of preformed liposomes is most commonly used for attaching large targeting molecules to the liposomes, such as proteins. It is important to realize that the attachment of large targeting molecules may cause a decrease in their targeting efficiency due to conformational changes [56–58]. In addition, the close proximity of the targeting molecule to the liposome bilayer may alter the stability of the liposomes. This problem may be avoided by using longer PEG moieties containing the functional groups. It has been shown that the length of the PEG moiety may influence the targeting efficiency most likely due to steric reasons [59].

A variety of different methods for coupling targeting ligands to the surface of preformed liposomes will be described.

#### 2.2.1. Classical Reactions to Modify Preformed Liposomes

**Figure 1:**
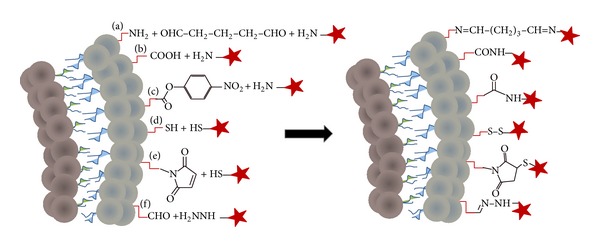
Classical methods for coupling ligands. Schematic representation of a cross section of a liposome bilayer containing functional groups to illustrate (a) crosslinking of primary amines by glutaraldehyde, (b) carbonyl-amine bond formation, (c) amide bond formation by the reaction of* para*-nitrophenylcarbonyl with primary amine, (d) disulfide bond formation, (e) thioester bond formation by the maleimide-thiol addition reaction, and (f) hydrazone bond formation. Coupling ligands are represented by red stars.

The approaches based on classical reactions such as amide bond formation, crosslinking amines through homobifunctional linkers, thioester bond formation by the maleimide-thiol addition reaction, disulfide bridge, and hydrazone bond formation are summarized first (Figure 1).

*(1) Amine-Functionalized Liposomes.* The natural lipid phosphatidylethanolamine (PE; amine-containing lipid) was used in early studies to functionalize liposomes, aimed at exploring the preparation of immunoliposomes for intravascular targeting [31]. The amine-functionalized liposomes contained lecithin, cholesterol, and PE in a 6 : 2 : 2 molar ratio and are reactive to amine-containing ligands using a linker (Figure 1(a)). A common approach is the use of imidoester or imine crosslinking by reacting dimethyl suberimidate or glutaraldehyde, respectively, with the amine-functionalized liposome and the targeting ligand that also contains an amine group. A variety of antibodies have been coupled to the surface of the nanocarrier using this crosslinking approach [31]. More relevant, the incubation of antibody-targeted liposomes (loaded with Indium-111 chloride) with substrate-coated matrices revealed that the antibodies coupled to liposomes preserve their affinity, specificity, and targeting towards the correct antigen [56]. However, a major disadvantage of this crosslinking approach is the homobifunctionality of the linkers that are used in large excess, leading to side reactions and loss of targeting ligand.

*(2) Carboxylic Acid-Functionalized Liposomes.* Postmodification of the liposome surface by amine-carboxyl conjugation (Figure 1(b)) was recently reported to overcome the rapid systemic clearance of D-phenylalanyl-L-prolyl-L-arginyl-chloromethyl ketone short peptide (PPACK), an antithrombin agent [30]. Palekar et al. [30] demonstrated that PPACK peptide is attached to the surface of preformed liposomes composed of EPC, DPPE, and DSPE-PEG_2000_-COOH (94 : 4 : 2 molar ratio) by applying standard amine-carboxyl coupling conditions for conjugating the N-terminus of the peptide to preformed carboxy-terminated DSPE-PEG-containing liposomes. The peptide was conjugated to the unilamellar liposomes and liposomes were purified by dialysis for four hours, which suggests high stability of the nanosystem. Attachment of the peptide to the liposome surface was confirmed by the zeta potential changing from −31.92 mV to −13.45 mV. The thrombin-targeted liposomes showed an improvement of the antithrombin effect; nevertheless, the systemic action of the targeted liposomes decreased after dosing, because high levels of liposomes accumulated in the liver and spleen 2 h after the injection [30]. Even though the half-life of the new antithrombin liposomal system is not reported, the results from the* in vivo* studies suggest a huge increase over the 3 min half-life of free PPACK peptide.

*(3) para-Nitrophenylcarbonyl-Functionalized Liposomes.* A decade ago Torchilin et al. introduced alternative methodology to conjugate proteins to liposomes in a highly effective single step [32]. A new DOPE derivative,* para*-nitrophenylcarbonyl (pNP)-PEG-DOPE was synthesized and used as component to prepare pNP-appended liposomes (Figure 1(c)). The lipid films for preparing the liposomes contained EPC and cholesterol in a 7 : 3 molar ratio with various amounts of (pNP)-PEG-DOPE and were hydrated in citrate buffer (pH 5.1) to prevent hydrolysis of the pNP moiety. The pNP moiety reacts with primary amines in aqueous buffer in the pH-range 8.0–9.5, yielding the desired liposome-protein nanosystem. This new lipid displays several advantages; first, only 0.5 mol% of total lipid is required to link a sufficient number of targeting protein molecules, and second, the unreacted pNP molecules will be hydrolyzed avoiding undesired side reactions. Moreover, no activation of the carboxylic acid is required simplifying the reaction and decreasing the overall time required for the surface modification [32]. On the other hand, the pNP-lipid must be maintained at acidic pH during its manipulation to prevent the hydrolysis of the pNP group. To assess the stability of the pNP-appended liposomes before and after protein ligation, calcein-loaded liposomes were incubated in mouse serum. Calcein was encapsulated in the liposomes at a self-quenching concentration, and the dequenching of calcein fluorescence due to leakage was determined at several time intervals [60]. Liposomes containing pNP-PEG-DOPE showed very similar levels of stability in mouse serum before and after linkage to the monoclonal antibody 2C5, whereas the liposomes without pNP-PEG-DOPE lipid were less stable under the same conditions [32]. In another report, TAT peptide was successfully conjugated to preformed liposomes containing pNP-PEG-PE lipid [61]. However, the stability of this system was not reported, and neither was the zeta potential of the peptide-conjugated liposomes, which may be relevant given the high positive charge of the TAT peptide. Conjugation of the TAT peptide or the monoclonal antibody 2C5 to the liposomes preserved the specific activity of the ligands [32, 61]. The possibility of altering the active conformation of the targeting ligand due to conjugation to liposomes is an important parameter to consider in the design and characterization of targeted liposome nanosystems [56–58].

**Figure 2:**
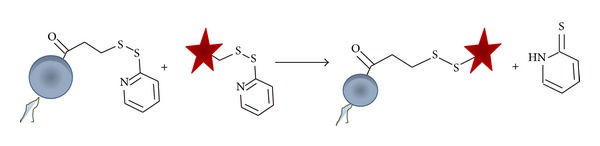
Schematic representation of the reaction between SPDP-modified PE (blue sphere) and a 2-pyridyldithio-modified targeting ligand (red star), leading to disulfide bond formation and the release of 2-thiopyridone.

*(4) Thiol-Functionalized Liposomes. *A protein-bound dithiopyridine (protein-DTP) can be activated at acidic pH to react with another dithiopyridine-modified molecule to form a disulfide bridge (Figure 1(d)), which provides the opportunity to use the DTP group for conjugating a protein to the surface of liposomes containing thiol-reactive moieties. An advantage of this coupling method is that the reaction can be monitored spectroscopically by the release of the chromophore 2-thiopyridone (Figure 2) [62].

Commonly, 3-(2-pyridyldithio)propionate (SPDP) is conjugated to PE yielding the desired DTP-phospholipid [43]. Liposomes targeted with Fab' antibody fragments appeared to be stable at physiological pH for longer than 7 h; nevertheless, the stability in serum was decreased, most likely due to the presence of biological reductants reducing the disulfide bond and releasing the antibody from the surface of the liposomes [62].

*(5) Maleimide-Functionalized Liposomes.* Maleimide- or bromoacetyl-functionalized DSPE was prepared and used to prepare liposomes also containing PC, PG, and cholesterol (10 : 65 : 25 : 50 molar ratio) [63]. Both the maleimide and the bromoacetyl moiety are reactive towards thiol-containing ligands. The conjugation of thiol-containing molecules to liposomes containing DSPE-maleimide and DSPE-bromoacetyl derivatives was investigated in detail by Schelté et al. [63]. It was found that maleimide reacts with thiols at pH 6.5 while bromoacetyl is reactive at pH 9.0, suggesting that both functionalities could be combined at the liposome surface to react with different thiol-containing ligands.

The commercially available DSPE-PEG-maleimide phospholipid has been widely used to functionalize liposomes with thiol-containing ligands (Figure 1(e)) [33–36]. More recently, monomeric and tetrameric H2009.1 peptides for targeting integrins *α*_*v*_ *β*_6_ expressed on non-small cell lung cancer cells were attached to the surface of preformed liposomes containing DSPE-PEG_2000_-maleimide [45]. Three liposome formulations varying in the concentration of DSPE-PEG-maleimide were investigated. For this purpose, three lipid films were prepared from chloroform : methanol solutions of HSPC and cholesterol (65 : 32 molar ratio) with DSPE-PEG_2000_ and DSPE-PEG_2000_-maleimide added at molar ratios of 2.5 : 0.64, 1.9 : 1.3, or 1.2 : 2. The lipid films were hydrated with ammonium sulfate buffer pH 5.5 at 65°C, the resulting liposomes extruded through a 100 nm pore size filter, and next the buffer was exchanged to citrate buffer at the same pH to allow for doxorubicin loading. Subsequently, the peptides (monomeric and tetrameric) with a C-terminal cysteine were conjugated to the liposome surface through the maleimide-thiol coupling reaction using an excess of the peptides in HEPES buffer. The efficiency of peptide coupling to the surface of the liposomes was 90% based on the amount of maleimide exposed on the liposome surface [45]. To study the localization of the peptide on the surface of the liposomes, the maleimide moiety on the liposomes was quenched by *β*-mercaptoethanol before the addition of the peptide. Once the maleimide moiety has reacted with *β*-mercaptoethanol, the peptide is not able to conjugate to the preformed liposomes. After an overnight incubation the peptide was not associated with the liposomes, confirming that the peptides are not spontaneously inserted into the lipid bilayer, which is highly important for recognition by the integrins *α*_*v*_ *β*_6_ on the cell membrane. The effect of the charge of monomeric (+2 charge) and tetrameric (+8 charge) peptides on the liposomes was investigated in* in vitro* experiments, because a higher concentration of peptide may lead to higher levels of liposome uptake due to their high positive charge. The* in vitro* experiments suggested a nonspecific cellular accumulation of the H2009.1-conjugated liposomes, consistent with the effect of the charge on the cellular uptake. To investigate specific integrins *α*_*v*_ *β*_6_ binding of the H2009.1 tetrameric peptide-conjugated liposomes, the authors tested a scrambled control peptide, scH2009.1, which is unable to bind to the integrins *α*_*v*_ *β*_6_, while containing the same amino acids and carrying the same charge as the H2009.1 peptide. The tetrameric peptide H2009.1-conjugated liposomes did display increased integrins *α*_*v*_ *β*_6_-specific cell binding as compared to scH20009.1 tetrameric peptide conjugated to liposomes, showing a receptor-specific binding effect in combination with nonspecific binding due to the peptide charge. The effect of the peptide charge on the zeta potential of these liposomes was not reported. Moreover, the occurrence of leakage of doxorubicin upon peptide binding via the maleimide-thiol reaction was not reported. Nevertheless, fluorescence microscopy on cells expressing integrins *α*_*v*_ *β*_6_ (H2009 lung cancer) suggested that the liposomes remained intact extracellularly, with release of doxorubicin only once they were taken up by the cells, suggesting insignificant leakage of the agent under physiological conditions.

*(6) Aldehyde-Functionalized Liposomes.* In the early 2000s Bourel-Bonnet et al. reported a novel method for ligation based on the formation of a hydrazone bond between an *α*-oxoaldehyde palmitoyl derivative and *α*-hydrazine acetyl peptides (Figure 1(f)) [44]. This approach presents several advantages. First the reaction proceeds under mild conditions with the generation of water and no undesired side products. Second, because natural macromolecules do not contain the functional groups that form the hydrazone bond, the ligation is regioselective. A more comprehensive study on the thermodynamic and kinetic parameters of the hydrazone ligation on liposomes was subsequently reported [37], which confirmed that the ligation is quantitative and selective. Moreover, when this ligation was performed in colloidal media under physiological conditions the kinetics and stability were enhanced due to autoassociation of the reagents, whereas the hydrazone bond displays lower stability in solution in the absence of colloidal media.

Several peptidoliposomes prepared using hydrazone ligation between aldehyde-functionalized liposomes and hydrazine acetyl peptides were investigated, confirming the utility of receptor targeting using biologically relevant peptidoliposomes [64].

#### 2.2.2. Recent Approaches

**Figure 3:**
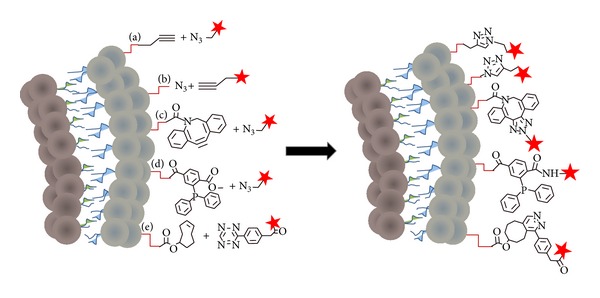
Bioorthogonal chemistry approaches for coupling ligands. Schematic representation of a cross section of a liposome bilayer containing functional groups to illustrate: (a) and (b) copper(I)-catalyzed Huisgen 1,3-dipolar cycloaddition (CuAAC) ligation, (c) copper-free click chemistry ligation, (d) the Staudinger ligation, and (e) tetrazine/*trans*-cyclooctene inverse electron demand Diels-Alder cycloaddition (IEDDA). Coupling ligands are represented by a red star.

The bioorthogonal chemistry approaches developed over the last decade display a wide impact on diverse research fields [65–68]. These chemical reactions feature chemoselectivity, mild reaction conditions in aqueous media, and good yields and, most importantly, the ligation occurs between functional groups that are not present in natural macromolecules, avoiding side products. From the early 2000s researchers have investigated the use of the so-called click chemistry to attach targeting ligands to the surface of liposomes [47, 48, 51]. Nowadays several click chemistry approaches are used for the ligation of ligands on the surface of liposomes. Four different types of click chemistry reactions have been exploited to modify the liposome surface (Figure 3), which are described below and illustrated with recent examples. In addition, enzymatic ligation of proteins to preformed liposomes and the His-tag chelating strategy for modifying the liposome surface will be described.

*(1) Copper(I)-Catalyzed Huisgen 1,3-Dipolar Cycloaddition (CuAAC).* Hassane et al. reported the first example of mannose ligation on the surface of liposomes, using copper-catalyzed click chemistry (Figure 3(a)) [51]. The authors investigated the optimal reaction conditions for conjugating an *α*-D-mannosyl azido derivative to alkyne-functionalized small unilamellar vesicles (SUV). The alkyne-functionalized liposomes contained DPPC, DPPG, cholesterol (70 : 20 : 50 molar ratio), and the alkyne-derivative N-[2-(2-(2-(2-(2,3-Bis (hexadecyloxy)propoxy)ethoxy)ethoxy)ethoxy)ethyl]hex-5-ynamide at 5–10 mol%. Ligation was successful with up to 80% yield with respect to the mannose after 1 h; copper(I) chelators however were found to be required to reduce aggregation of the vesicles and decrease reaction time. The stability of the vesicles during the ligation reaction was confirmed using 5,6-carboxyfluorescein (CF). Like calcein, 5,6-carboxyfluorescein is a self-quenched fluorophore when entrapped in liposomes at high concentration and is commonly used to study the stability of liposomes by fluorescence spectroscopy [69]. When CF is released from the liposomes, the fluorescence emission of the dye increases, providing information on the (in)stability of the system [69].

Azido-functionalized liposomes for the ligation of peptides on the vesicle surface using copper-catalyzed click chemistry were also investigated (Figure 3(b)) [49, 50]. The alkyne function was introduced at the C-terminus of the gH625 peptides and subjected to click chemistry with azide-AdOO-Lys(C(O)CH_2_CH_2_C(O)N-(C_18_H_37_)_2_) incorporated in the liposomal membranes. A similar approach was followed more recently by the same research group to conjugate tetrabranched neurotensin peptides ((NT8-13)_4_-alkyne) with doxorubicin remote-loaded liposomes [50]. In both studies the conjugation was performed overnight in HEPES buffer using copper(I) catalyst generated in situ from a mixture of CuSO_4_ and ascorbic acid. The yield of the click reaction of the gH625 peptide to the azido-liposomes was 90% with respect to the peptide, whereas 95% yield was obtained for the ligation of (NT8-13)_4_-alkyne. The use of a copper chelating agent to shorten the reaction time was not discussed.

Despite the success of the CuAAC approach, the copper catalysts required for the reaction to occur may lead to complications due to the toxicity of copper(I) ions.

*(2) Copper-Free Click Chemistry.* Problems with the use of copper(I) in biological systems has resulted in the development of a copper-free click chemistry reaction [70]. This reaction is also known as the “ring-strain promoted” reaction due to the fast reactivity of a cyclooctyne ring with an azido group forming a triazole ring (Figure 3(c)). The main advantage of this class of click chemistry is that no catalyst is required.

Koo et al. explored the use of copper-free click chemistry for* in vivo* targeting of liposomes to cancer cells [48]. First, DSPE-PEG-DBCO lipid (DBCO stands for dibenzocyclooctyne) was prepared using standard amide bond formation between DSPE-PEG-NH_2_ and sulfo-NHS-DBCO. Subsequently, DBCO-liposomes were prepared using a lipid film composed of DPPC : cholesterol : DBCO-PEG-DSPE : Cy5-DPPE (54.5 : 35 : 10 : 0.5 molar ratio), which was hydrated with PBS. To conjugate the liposomes on the surface of the tumor cells by copper-free click chemistry, the authors used the same approach as Mahal et al. [71], who previously engineered cell surface exposed oligosaccharides with different functional groups through the biosynthesis of sialic acid in cells. The A549 tumor cells were treated with tetraacylated N-azidoacetylmannosamine (Ac_4_ManNAz) and after several days displayed sialic acids with an appended azido group on the cell surface, as previously reported [72, 73]. Successful attachment of the DBCO-liposomes to the cell surface via copper-free click chemistry was accomplished, and subsequent intracellular uptake of the liposomes by the tumor cell was observed. In addition, mice were treated with Ac_4_ManNAz by intratumoral injection, three days before DBCO-liposomes were administered intravenously. The results from these* in vivo* studies and the analysis of the tumor tissues suggested that the DBCO-liposome is delivered to the targeted tumor* in vivo*. Drug delivery, however, was not attempted.

*(3) The Staudinger Ligation.* The reaction between a phosphine and an azide producing aza-ylide was discovered by and named after Staudinger [74]. However, the formed aza-ylide hydrolyzes in water, yielding an amine and phosphine oxide and dissociating the ligation product. In order to exploit the Staudinger reaction in aqueous, biological systems, Saxon and Bertozzi introduced an electrophilic trap in the form of a methyl ester group in the* ortho* position to the diphenylphosphine derivative, which ultimately captures the aza-ylide intermediate by cyclization [72]. As a result an amide bond between the phosphine derivative and the azide derivative is formed. This method is nowadays widely used for bioorthogonal ligation.

The Staudinger ligation was only recently explored to modify the surface of liposomes (Figure 3(d)) [47]. A terminal triphosphine-derivatized lipid was prepared by amide coupling of DPPE and diphenylphosphino-4-methoxycarbonylbenzoic acid. Liposomes were prepared containing DPPC and cholesterol (2 : 1 molar ratio) with 5 mol% of the triphosphine-DPPE derivative. The lipid film was hydrated with PBS resulting in multilamellar vesicles, which were freeze-thawed and extruded to obtain large unilamellar vesicles. To demonstrate the reactivity of the triphosphine moiety towards azide groups, the triphosphine-liposomes were subsequently mixed with an azide-containing lactose ligand to prepare lactosylated liposomes. An 80% functionalization of the triphosphine-liposomes, based on carbohydrate quantification using phenol-sulfuric acid, was obtained, based on the assumption that about 40% of the triphosphine moiety is in the outer leaflet on the liposomes [47].

The stability of the liposomes during the ligation was confirmed by the absence of significant leakage from carboxyfluorescein-entrapped liposomes under the ligation conditions. In addition, aggregation of the lactosylated liposomes over time was evaluated and compared to those without lactose ligand using dynamic light scattering. Particle stability turned out to be improved by the lactose “coating.”

The Staudinger ligation has been demonstrated to exhibit high chemoselectivity and is compatible with biological processes [73]. Moreover, like copper-free click chemistry, the Staudinger ligation has the advantage of being a catalyst-free reaction. The major disadvantage of the triphosphines is their slow oxidation to phosphine oxide, which will halt the cyclization and consequently the ligation with the azido-containing molecule [47]. However, the oxidation is slow and can be minimized by using an inert atmosphere during the manipulations of the triphosphine derivative [47].

*(4) Tetrazine/Trans-Cyclooctene Inverse Electron Demand Diels-Alder Cycloaddition (IEDDA).* Several years ago Blackman et al. described a fast and new bioorthogonal chemical reaction using the reactivity of* trans*-cyclooctene (TCO) with a tetrazine ring (Figure 3(d)) [75]. This reaction is based on the inverse electron-demand Diels-Alder reaction (IEDDA) of the dienes of the tetrazine ring with the dienophile TCO, upon retro-[4 + 2] cycloaddition. Several studies have confirmed the utility of this chemistry* in vivo* [76, 77].

Very recently, ^18^F-labeled liposomes have been applied in* in vivo* targeting using IEDDA [52]. The authors envisioned the preparation of radiolabeled liposomes with* trans*-cyclooctene on their surface for bioorthogonal ligation to tetrazine-modified tissues. A new lipid containing* trans*-cyclooctene was prepared using DSPE-PEG_2000_-amine and TCO-NHS ligand [52]. The liposomes contained DSPC/cholesterol/DSPE-PEG_2000_/DSPE-PEG_2000_-TCO (65/30/2.5/2.5) labeled with [^18^F]fluoro-1,2-dipalmitoyl glycerol and were prepared by sonication and extrusion to obtain a size distribution around 143 nm. The cyclooctene moiety was found to retain reactivity after liposome formation. In order to visualize tumor tissues with the radiolabeled liposomes using IEDDA chemistry in mice, the tetrazine ring must first be incorporated in the tumor tissue. A linear peptide (pHLIP) was conjugated to the tetrazine ring by maleimide-thiol reaction between a maleimide-tetrazine derivative and the cysteine terminated pHLIP peptide and then injected into the tumor tissue of the mice. The peptide pHLIP changes to an *α*-helical conformation in the acidic environment of the tumor, which allows the tetrazine-pHLIP derivative to be inserted into the membrane of the cancer cells [78]. Once the tetrazine moiety is exposed on the surface of the cell membrane, the ^18^F-radiolabeled TCO-liposomes are able to conjugate to these cells, which was monitored by positron emission tomography (PET) [52]. Only 30 min after intravenous injection of the ^18^F-TCO-liposomes, the tumors were visible by PET. A vast variety of control studies were performed, which confirmed the success of the bioorthogonal coupling between the ^18^F-TCO-liposomes and the tetrazine-modified tissue. Nevertheless, 1 h after administration, liposomes could be found in the liver (12.8% injected dose/gram) and the intestines (16% injected dose/gram). The* in vitro* stability of the tetrazine moiety was assayed, yielding a 29 h half-life in serum. Overall, this elegant study has demonstrated the successful use of IEDDA chemistry with appropriate pharmacokinetics for* in vivo* therapeutic or imaging agent delivery applications.

*(5) Enzymatic Modification of Liposome Surface.* All the strategies described above have proven highly useful for the modification of liposomes. More innovative new approaches, however, continue to be developed. Recently, Guo et al. reported a novel approach to functionalize liposomes using the enzymatic activity of sortase A [79]. Sortase A is a transpeptidase that is able to recognize peptide LPXTG at the C-terminus of a protein (amino acid X is variable) [80]. The enzyme cleaves the peptide bond between T (Thr) and G (Gly) forming a thioester bond with threonine (Thr) [81]. Subsequently, the free carboxylic group of Thr is transferred to the substrate (peptidoglycan) which displays a Gly at the N-terminus, thus forming a new peptide bond, the so-called transpeptidation product. With this reactivity in mind, the authors envisioned the modification of the liposome surface with LPXTG-containing proteins using sortase A transpeptidation activity. For this purpose, enhanced green fluorescent protein (eGFP) containing LPATG-H_6_ peptide at the C-terminus was expressed in* E. coli*. In addition, two different lipids containing a glycine residue with a free N-terminus were prepared, DSPE-GG-NH_2_ and DSPE-PEG_2000_-GG-NH_2_. These lipids were incorporated at 2 mol% of total lipids in liposomes (DSPC/cholesterol, 2 : 1 molar ratio) prepared by extrusion after hydration of the lipid film with Tris-buffer. Conjugation of the liposomes with eGFP-LPATG-H_6_ was performed using sortase A overnight at 37°C. SDS-PAGE and fluorescence imaging confirmed the conjugation of eGFP to the liposomes, which was more efficient for the liposomes containing reactive glycine attached to the PEG_2000_ spacer [79], most likely due to steric reasons. Even though this site-specific modification of liposomes is very promising, the authors did not perform stability studies after the enzymatic reaction to ligate the protein to the liposome surface. Preservation of liposome stability during ligation is crucial for the application of liposomal functionalization with biological macromolecules.

*(6) His-Tag Chelating Strategy on Liposome Surface.* Limitations in delivering antigens into dendritic cells with significant effect on the immune response* in vivo* encouraged van Broekhoven et al. to develop a new strategy [82]. For this new approach a chelator lipid (NTA_3_)-DTDA (three nitrilotriacetic acid moieties on ditetradecylamine) was synthesized and used to prepare antigen containing liposomes. The chelator acts as an anchor for histidine-tagged single chain full-length variable Ab fragments (ScFv) to be attached to the liposome surface [82]. Liposomes were prepared from a lipid film composed of POPC, NTA_3_-DTDA, PE-PEG_2000_, LPS (lipopolysaccharide for interaction with dendritic cells), and Bodipy-PC (96 : 1 : 1 : 1 : 1), which was hydrated with PBS containing Ni^2+^. The nickel ions allow the anchoring of different His-tagged molecules on the surface of the liposomes by the Ni(II) metal ion-chelating effect of the NTA groups. Based on the effective dissociation constant of three NTA moieties being 5- to 10-fold lower than for a single NTA chelating moiety, the authors hypothesized that NTA_3_-liposomes would efficiently anchor histidine-tagged single chain full-length variable Ab fragments against dendritic cell markers. Stable engraftment of His-tagged ScFv specific for markers CD11c and DEC-205 was accomplished and proven successful* in vitro* and* in vivo* [82]. This pioneering study confirmed antigen delivery by NTA_3_-DTDA liposomes as an alternative to the* ex vivo* dendritic cells manipulations. This strategy has successfully been applied for preparing targeted liposomes by several authors [83–87].

More recently, the chelator NTA_3_-DTDA was explored for anchoring histidine-tagged targeting peptides on the surface of doxorubicin-loaded liposomes [87]. The authors optimized the postinsertion of NTA_3_-DTDA in commercially available doxorubicin-loaded liposomes. NTA_3_-DTDA postinsertion was performed by incubating the liposomes with 1% NTA_3_-DTDA (with respect to total lipid) at 37°C for 2 h. No significant leakage of the drug was observed after size-exclusion purification, and the liposomal size distribution was almost not altered after NTA_3_-DTDA postinsertion. Two tumor-homing peptides were investigated for targeting purposes, p15-RGR and p46-RGD, both containing a polyhistidine-tag on their N-terminus, targeting PDGFR*β* [88] and *α*_*v*_-integrins [89], respectively. The anchoring of the peptide was performed in aqueous media containing NiSO_4_, the NTA_3_-DTDA-liposomes, and the His-tagged peptides for 30 min. The peptide-engrafted doxorubicin liposomes were studied* in vitro* and* in vivo*. In particular, the p15-RGR-engrafted doxorubicin liposomes showed increased cytotoxicity against NIH-3T3 cells (murine fibroblast) as compared to control liposomes. Nevertheless, the biodistribution of the liposomes in mice did not show significant accumulation in the B16-F1 tumor, and no effect on tumor growth was detected. In contrast, the p46-RGD-engrafted doxorubicin liposomes were found to accumulate in subcutaneous B16-F1 tumors in mice. The poor efficacy of the p15-RGR peptide-engrafted liposomes towards tumor B16-F1 was attributed to differences in PDGFR*β* expression levels in the tumor model used in this study.

The polyhistidine-tag is commonly fused to the proteins for ease of purification. Modification of the liposome surface with three nitrilotriacetic acid (NTA_3_) provides the opportunity to modify liposomes with a vast variety of His-tagged proteins (or peptides). The stability of the liposomes may however be compromised depending on the nature of the protein.

## 3. Conclusions and Perspectives

The delivery of active agents using liposomes as nanocarriers has made significant advances over the last four decades, with several formulations in clinical application and trials [9]. The lessons learned during many years of multidisciplinary research have been extensively reviewed [6–8]. Given the success of liposome-based drug delivery, it is evident that these systems have a bright future in pharmaceutical applications, particularly with the increasing availability of novel strategies for targeting the liposomes to the diseased tissue. This review summarized some of these approaches. More common ligation strategies such as amine-carbonyl ligation, disulfide bridge bond formation, thioester bond, and hydrazone bond were developed several decades ago and are widely used [7]. Nevertheless, these strategies have several drawbacks including undesired side products and promiscuous reactivity. With the development of bioorthogonal chemistry, new strategies to modify the liposome surface have been explored. Starting from copper(I)-catalyzed click chemistry, it was shown that bioorthogonal chemistry is a powerful alternative to ligate ligands to the surface of liposomes [49–51]. The toxicity of copper(I) led to the development of copper-free click ligation strategies. Both DBCO and triphosphine groups react with azido-containing ligands to give the ligation product in high yield under mild reaction conditions [47, 48]. The subsequently developed tetrazine/*trans*-cyclooctene inverse electron demand Diels-Alder cycloaddition (IEDDA) ligation strategy was also applied to successfully target liposomes to tumor tissue [52]. It is clear that the development of new strategies to ligate ligands to the surface of liposomes reflects the state-of-the-art of biocompatible chemical ligation strategies with increased efficiency. The bioorthogonality of the click reactions, their high yield, and mild reaction conditions render them the most promising strategies for ligating ligands to liposomes.

Other promising innovative approaches for ligating ligands to the liposome surface include transpeptidation catalyzed by sortase A [79] and the chelating strategy employing His-tagged proteins and nitrilotriacetic acid (NTA_3_) modified liposomes [84–86]. The latter strategy has opened the opportunity to modify liposomes with a vast variety of His-tag possessing proteins (or peptides). However, this strategy was found to have limitations in eliciting antibody response as compared to covalent binding of the protein to the liposome [90].

Even though advantages and disadvantages can be pointed out for each ligation strategy, it can be concluded that the targeting of liposomes has strongly improved in the last decade. It remains to be proven whether active targeting will be successful in clinical applications. Multidisciplinary research will remain required to find the optimal liposomal formulation for a given therapeutic agent and to optimize the ligation strategy for a given targeting ligand.
